# Metabolic Fitness of T Cells in Autoimmune Disease

**DOI:** 10.20900/immunometab20200017

**Published:** 2020-04-22

**Authors:** Bowen Wu, Jörg J. Goronzy, Cornelia M. Weyand

**Affiliations:** 1 School of Medicine, Stanford University, Stanford, CA 94305, USA; 2 Department of Medicine, Palo Alto Veterans Administration Healthcare System, Palo Alto, CA 94304, USA

**Keywords:** rheumatoid arthritis, systemic lupus erythematosus, T cells, glucose, glycolysis, mitochondria, oxygen consumption, lipid droplets, lipogenesis

## Abstract

Rheumatoid arthritis (RA) and systemic lupus erythematosus (SLE) are relatively common autoimmune diseases, often considered prototypic examples for how protective immunity switches to destructive immunity. The autoantigens recognized in RA and SLE are distinct, clinical manifestations are partially overlapping. A shared feature is the propensity of the adaptive immune system to respond inappropriately, with T cell hyper-responsiveness a pinnacle pathogenic defect. Upon antigen recognition, T cells mobilize a multi-pranged metabolic program, enabling them to massively expand and turn into highly mobile effector cells. Current evidence supports that T cells from patients with RA or SLE adopt metabolic programs different from healthy T cells, in line with the concept that autoimmune effector functions rely on specified pathways of energy sensing, energy generation and energy utilization. Due to misrouting of the energy sensor AMPK, RA T cells have a defect in balancing catabolic and anabolic processes and deviate towards a cell-building program. They supply biosynthetic precursors by shunting glucose away from glycolytic breakdown towards the pentose phosphate pathway and upregulate lipogenesis, enabling cellular motility and tissue invasiveness. Conversely, T cells from SLE patients are committed to high glycolytic flux, overusing the mitochondrial machinery and imposing oxidative stress. Typically, disease-relevant effector functions in SLE are associated with inappropriate activation of the key metabolic regulator mTORC1. Taken together, disease-specific metabolic signatures in RA and SLE represent vulnerabilities that are therapeutically targetable to suppress pathogenic immune responses.

## INTRODUCTION

Rheumatoid arthritis (RA) and systemic lupus erythematosus (SLE) are two representative autoimmune diseases that affect large patient populations and display typical features of immune system abnormalities. Both diseases can cause a broad spectrum of organ pathology. In RA, the main manifestation of auto-aggression is a chronic-persistent, symmetrical and destructive joint inflammation, while patients affected by SLE can have inflammatory injury of almost every organ system. RA and SLE share in common that affected individuals have a multi-fold higher risk to develop cardiovascular complications [1–3]; often understood as a complication of chronic-smoldering inflammation. Cardiovascular risk imposed by long-standing autoimmunity suggests that deficiencies of tissue homeostasis and repair may be part of the overall immune defect.

For most autoimmune diseases, the breakdown of self-tolerance and autoantibody production precedes clinical onset of disease by years to decades [4,5], indicating a fundamental and chronic rewiring of the immune system. HLA class II-restricted CD4 T cells, major players in inflammatory lesions and indispensable to induce and sustain autoantibody production by B cells, are central drivers in autoimmune diseases [6]. T cells rely strongly on bio-energetic and biosynthetic plasticity to fulfill their functions. With the ability to massively proliferate and commit to lineage differentiation, to invade and reside in different tissue microenvironments, such T cells encounter heterogeneous metabolic demands. Thus, the metabolic machinery is vulnerable to even minor changes translating into major, unexpected outcomes. In this review, we will summarize metabolic features of T cells in both RA and SLE patients; identifying metabolic checkpoints where T cells are mistakenly led into distinct disease-promoting directions.

## GLUCOSE UTILIZATION IN RA AND SLE T CELLS

With fatty acids as the main fuel during the naive state [7], effector T cells exhibit a dramatic metabolic switch towards glycolysis upon activation, upregulating glucose transporters and a large spectrum of glycolytic enzymes [8,9]. In contrast, regulatory T cells and memory T cells depend mostly on fatty acid oxidation [7,10] to support their survival needs and their functional activities. As a rule, maintenance of T cell nativity, memory and regulatory function is closely linked to lipid utilization and thus is ultimately dependent on mitochondrial fitness [11], whereas effector T cells, including the pro-inflammatory T cells that sustain tissue inflammation, basically, fuel their energy demands through fast access to glucose and glycolysis.

**Figure 1.**
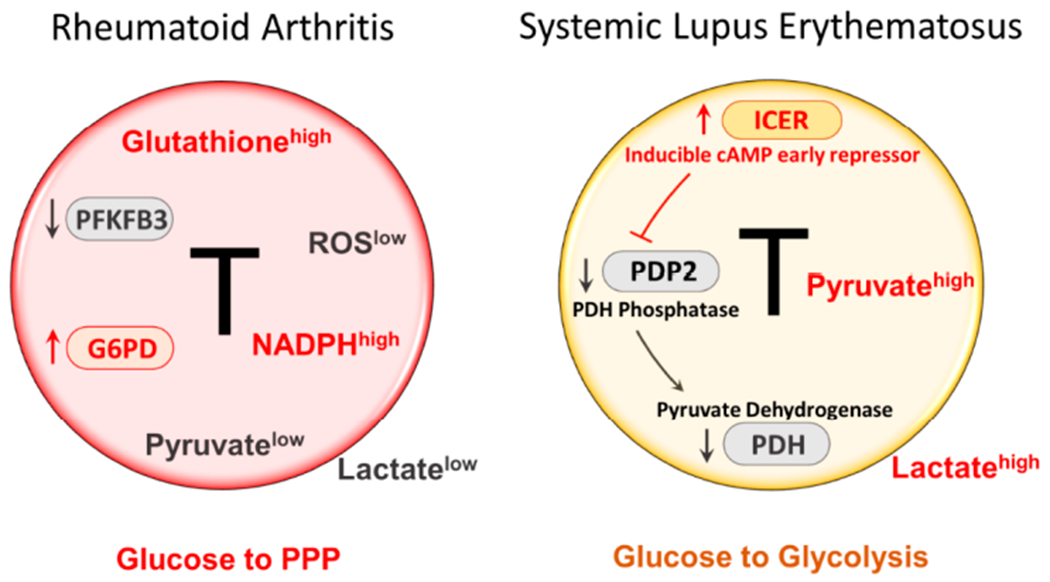
Rerouting of glucose utilization in RA and SLE T cells. RA T cells fail to upregulate PFKFB3, a key enzyme in the glycolytic pathway, leading to low production of pyruvate and lactate. Instead, RA T cells upregulate expression and function of G6PD, the enzyme that controls the entry to the pentose phosphate pathway (PPP). As a result, RA T cells produce more NADPH and shift the cellular redox balance towards a reductive environment (low ROS, high glutathione). T cells from SLE patients increase glycolytic breakdown of glucose and produce copious amounts of pyruvate and lactate. The intracellular environment is biased towards oxidative stress. One mechanism underlying the preferential routing of glucose towards glycolysis relates to the suppression of pyruvate dehydrogenase (PDH). An upstream event is the high expression of the transcription factor inducible cAMP early repressor (ICER), which, in turn, leads to inhibition of PDH, and suppression of PDH. ICER is considered a lineage-promoting transcription factor promoting Th17 commitment. Th17 cells are recognized as key effector cells in SLE. Overall, RA T cells are programmed towards anabolic activity, whereas SLE T cells favor catabolic metabolism. PPP, pentose phosphate pathway; ROS, reactive oxygen species; PDP2, pyruvate dehydrogenase phosphatase catalytic subunit 2.

Glucose is actively transported into the cell and is catalyzed into glucose-6-phosphate (G6P) by hexokinases (HKs). Subsequently, G6P either goes into glycolytic breakdown to produce pyruvate or enters the pentose phosphate pathway to generate NADPH and nucleotide precursors. A key event in the glycolytic breakdown is the phosphorylation of fructose 6-phosphate to fructose-1, 6-bisphosphate through 6-phosphofructo-l-kinase (PFK1), an irreversible reaction which commits glucose to glycolysis. PFK1 activity is dominantly controlled by its allosteric activator fructose-2, 6-bisphosphate (F2, 6BP), generated by 6-phosphofructo-2-kinase/ fructose-2, 6-bis-phosphatase-3 (PFKFB3). Hence, PFKFB3 critically regulates the glycolytic rate and PFKFB3 is the target of regulatory events that dictate the efficiency of cytoplasmic glucose utilization. T cells from RA patients fail to upregulate PFKFB3 upon activation (Figure 1), suppressing glycolytic breakdown and limiting pyruvate/lactate production [12,13]. On the contrary, RA T cells transcriptionally upregulate G6PD, shifting glucose into the pentose phosphate pathway (PPP) (Figure 1). The outcome of a dysbalance in the PFK/G6PD ratio is the overproduction of NADPH and accumulation of reduced glutathione, creating a reductive intracellular environment [14,15]. This fundamental change in redox homeostasis significantly disrupts proper sensing of the energy state, mistakenly promoting cell proliferation because of insufficient activation of the cell cycle checkpoint kinase ATM [12,16]. Most of these data have been collected in patient-derived CD4 T cells and the metabolic switch from catabolism and glucose breakdown to anabolism and generation of biosynthetic precursor is already present in the naive CD4 T cell population, and these metabolic features are specific for the T cell compartment [17]. It is therefore highly unlikely that these metabolic adaptations are the result of T cells living in an inflammatory tissue environment. Rather, the invasion of T cells into peripheral tissue sites, where they drive inflammatory pathways and set up tissue residence, is already a consequence of metabolic reprogramming.

Once CD4 T cells in RA patients have fully committed to effector cell differentiation, they utilize metabolic programs typical for effector T cells (reviewed in [7–11]).

The inflamed synovial lesions exclusively contain memory and effector T cells and these cells are exposed to metabolic cues imposed by the tissue environment. Besides having to share energy resources, lesional T cells will receive metabolic signals from neighboring cell populations, which are highly metabolically active [18]. A dominant metabolite encountered in the inflamed joint is lactate [19,20], a breakdown product of glucose, produced by metabolically active stromal cells, endothelial cells and invading immune cells. Lactate has been implicated in directly shaping T cell effector functions by increased IL-17 production via nuclear PKM2/STAT3 and enhanced fatty acid synthesis [21]. Also, lactate can inhibit the migration of both CD4 and CD8 T cells, thus causing T cell entrapment within the inflamed synovial tissue [22]. Elevated frequencies of ICOS^+^ Tfh cells have been described in the synovial fluid of RA patients, and targeting glycolysis in such Tfh cells has been reported to ameliorate established collagen-induced arthritis [23].

Fundamentally different from RA T cells, CD4 T cells from lupus patients and lupus-prone mice display high demand for glucose [24,25] and depend on rapid production of ATP, requiring both mitochondrial activity and cytoplasmic glycolysis [26]. Interestingly, in SLE T cells the pentose phosphate pathway appears to be also hyperactive, although the underlying mechanisms remain unclear [27].

Much of the focus in SLE has been on follicular helper CD4 T (Tfh) cells, which are critically involved in providing help for B cell maturation and autoantibodies production. In SLE patients, frequencies of circulating Tfh cells are elevated and such frequencies are often correlated with disease load [28]. Metabolic needs of autoreactive Tfh cells appear to be different from influenza virus-specific helper T cells [29]. In murine models of SLE, broad inhibition of glycolysis, achieved by treating with 2-deoxyglucose (2-DG), successfully suppressed the expansion of autoreactive Tfh cells, but seemed to not affect B cell responses to exogenous antigens nor the induction of anti-influenza Tfh cells. These findings suggest differential metabolic wiring and differential sensitivity to intervention of protective and tissue-inflammatory T cells.

Amongst T cell effector cells, Th17 cells stand out as pathogenic drivers in SLE. Patients with SLE have a higher frequency of Th17 cells, which contribute to the establishment of proinflammatory conditions by infiltrating multiple organs [30,31]. Therefore, the molecular machinery that induces Th17 differentiation is of high interest in the investigation of SLE pathogenesis. Inducible cAMP early repressor (ICER), an isoform of the cAMP Response Element Modulator (CREM), is exclusively induced in Th17 cells and promotes Th17 cell differentiation via binding to the IL-17 promoter region to recruit the canonical enhancer RORγt. Increased ICER concentrations in CD4 T cells from patients with SLE have been associated with Th17 cell differentiation and disease severity [32]. In recent studies, ICER has been implicated in yet another mechanism promoting Th17 differentiation, and thus enhancing risk for SLE [33]. Th17 cells favor glycolytic metabolism, preferentially using pyruvate for lactate generation instead of mitochondria-dependent oxidative phosphorylation. Pyruvate dehydrogenase (PDH) is the bifurcation enzyme controlling the balance between lactate production and oxidative phosphorylation, with its activity critically controlled by its phosphorylation status. Pyruvate dehydrogenase phosphatase (PDP) can dephosphorylate and activate PDH. In SLE T cells, high levels of ICER inhibit pyruvate dehydrogenase phosphatase catalytic subunit 2 (PDP2) expression, impairing PDH dephosphorylation and activation, ultimately funneling glycolytic breakdown of glucose towards lactate (Figure 1). Forced overexpression of PDP2 reduced Th17 differentiation in CD4 T cells from both SLE patients and lupus-prone mice. These elegant studies have given new insights into regulatory functions of ICER, and how it targets the metabolic machinery. ICER’s downstream targets PDP2/PDH are obvious checkpoints in Th17 differentiation and are possibly druggable to avoid the Th17 bias driving SLE pathology (Figure 1).

## MITOCHONDRIA DNA DAMAGE AND INFLAMMASOME ACTIVATION IN RA T CELLS

**Figure 2.**
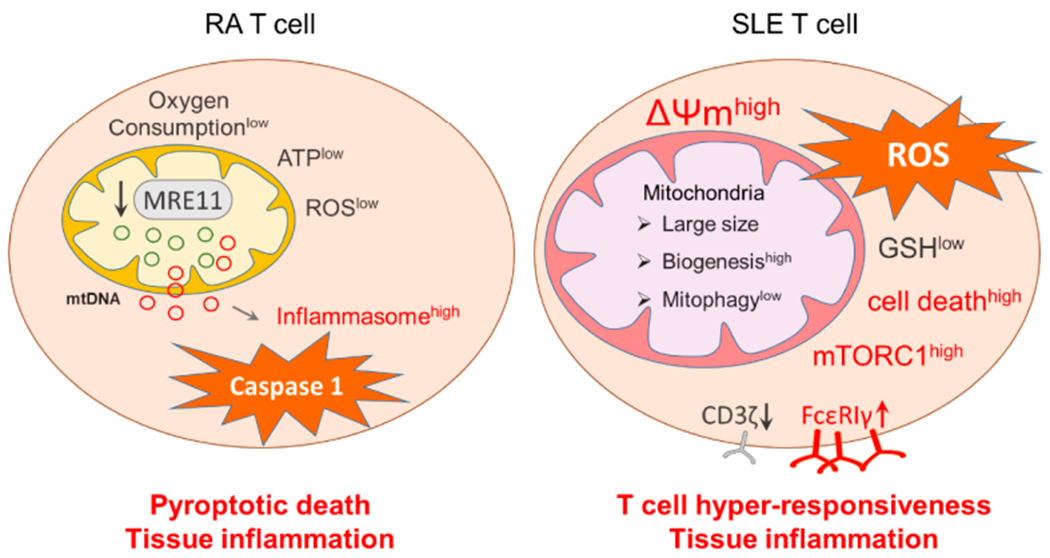
Mitochondria in RA and SLE T cells. RA T cells lose expression of MRE11, a nuclease functioning as a DNA repair molecule. Lack of mitochondrial MRE11A results in damage of mitochondrial DNA, increased susceptibility of mtDNA to oxidative damage and leakage of mtDNA into the cytoplasm. Cytoplasmic mtDNA is recognized as a danger associated molecular pattern (DAMP), initiates assembly of the inflammasome and activates caspase-1. As a result, T cells undergo pyroptotic cell death, release IL-1β and IL-18 and function as a nidus of tissue inflammation. In SLE T cells, mitochondrial biogenesis and fusion are enhanced while mitophagy is decreased. Thus, SLE T cells have high mitochondrial mass and elevated mitochondrial transmembrane potential [ΔΨm]. Inevitably, such T cells produce abundant ROS and exhaust cellular glutathione stores. The oxidative intracellular environment leads to cell death and drives mTORC1 activation. Downstream events of mTORC1 activation include recycling and lysosomal degradation of the T cell surface receptor/CD3ζ chain (TCRζ). Compensatory upregulation of the Fcε receptor type Iγ chain (FcεRIγ) and recruitment of tyrosine-protein kinase (Syk) enhance calcium fluxing, rendering these T cells hyper-reactive. MRE11, meiotic recombination 11; ROS, reactive oxygen species; GSH, glutathione; mTORC1, mammalian target of rapamycin complex 1.

Telomeres serve as sensors of cellular aging and CD4 T cells from RA patients have age-inappropriate shortening of telomeres [34,35], indicating that RA T cells are prematurely aged [36]. Deficiencies of the nuclear DNA repair machinery have been demonstrated to play a critical role in this pre-mature aging phenotype [37,38]. Besides nuclear DNA damage, a recent study reported that defects in DNA damage repair of RA T cells extend to mitochondrial DNA (mtDNA) [39]. Molecular studies have identified a loss-of-function of MRE11A, a nuclease and key component of the MRN complex [40]. MRE11A downregulation results from transcriptional repression, but upstream signals inducing the MRE11A^low^ phenotype have not been identified yet. Functional studies have revealed that MRE11A localizes to the telomeric ends but is also placed in the mitochondria. In RA T cells, the nuclease is expressed at significantly lower concentrations, rendering both telomeric ends and mitochondrial DNA susceptible to unrepaired DNA damage [39,40]. Functional consequences include persistent damage repair activity and in the case of mitochondria, loss of function and leakage of damaged mtDNA into the cytoplasm (Figure 2). Mitochondrial biogenesis appears unaffected by the reduction of mitochondrial MRE11A, as healthy control and RA T cells carry similar mitochondrial mass. Specifically, mitochondrial oxygen consumption is suppressed in RA T cells, suggestive for a defect in the mitochondrial electron transport chain.

Also, insufficient repair and maintenance of mtDNA resulted in the leakage of unrepaired and oxidatively modified DNA into the cytoplasm, where it was recognized by DNA sensors and triggered AIM2 and NLRP3-mediated inflammasome assembly and activation [39] (Figure 2).

The concept of implicating inflammasome activation in T cells in chronic tissue inflammation is novel. T cell loss in HIV infection has been attributed to inflammasome-dependent pyroptotic cell death [41]. Whether the inflammasome shapes function and survival of SLE T cells is currently unexplored. However, inappropriate inflammasome activity in myeloid cells is considered a critical disease pathway in SLE [42]. Inflammasome-related IL-18/IL-1β signaling has been associated with abnormal T cell responses, especially the Th17/Treg balance [43], which represent a new paradigm in inflammatory diseases. Both IL-18 and IL-1β have been reported to enhance Th17 differentiation while inhibiting the regulatory function of Treg cells [44,45]. The NLRP3 inflammasome is hyperactive in macrophages of SLE patients [46], creating a pro-inflammatory tissue microenvironment. Similarly, inflammasome activation contributes to dendritic cell function and affects their role as antigen-presenting cells; e.g., by fostering T cell activation and Th1 and Th17 polarization [47]. The immediate products of inflammasome activation, IL-18 and IL-1β, have been positively associated with disease activity in SLE [48,49]. Opposing findings have been reported in systemic lupus nephritis, where inflammasome activation appears to suppress excessive immunity. Loss-of-function of the NLRP3/ASC inflammasome in C57BL/6-lpr/lpr mice enhanced dendritic cell and macrophage activation, triggered massive lymphoproliferation, increased T cell infiltrates in the lung and resulted in severe proliferative lupus nephritis [50]. These data suggest a homeostatic role of inflammasome activation, participating in balancing protective and destructive immunity. Underlying mechanisms are insufficiently understood, but suppression of TGF-β target genes may be involved [50].

## MITOCHONDRIA HYPERPOLARIZATION AND ROS STRESS IN SLE T CELLS

A hallmark of CD4 T cells from SLE patients is mitochondrial hyperpolarization (MHP), measured as elevated mitochondrial transmembrane potential [ΔΨm] and reactive oxygen species (ROS) production [51,52] (Figure 2). In essence, lupus T cells are in a state of intense and lasting mitochondrial stimulation. Part of this increase in mitochondrial activity may be attributable to an increase in mitochondrial biogenesis [53], such that SLE T cells carry a higher load of mitochondria. Mitochondrial hyperactivity has numerous consequences for T cells, most prominently, the undermining of cellular defense mechanisms regulating oxidative stress. Glutathione, the most abundant intracellular antioxidant, is significantly reduced in SLE T cells, creating an oxidative intracellular environment. Multiple studies have corroborated that the mitochondrial building program is stimulated in SLE T cells. Large mitochondrial size, increased mitochondrial biogenesis and defective mitophagy are features of SLE T cells [53,54]. Upstream signals triggering the abnormal mitochondrial activation program remain elusive.

Downstream consequences of chronic mitochondrial stress have been carefully examined. As expected, the mammalian target of rapamycin (mTOR), a sensor of the mitochondrial transmembrane potential and ROS supply [55], is activated in lupus T cells [56]. The cellular growth program, in particular the manufacturing of proteins, depends on mTORC1 to sense nutrient availability, balance energy production and utilization, and direct energy carriers into proper pathways [57]. As part of this program, mTORC1 regulates the endocytic recycling machinery (Rab5A and HRES-1/Rab4 small GTPases), which determines the half-life and positioning of cell surface receptors, including the T cell surface receptor/CD3ζ chain (TCRζ) [56]. In lupus T cells, chronic mTORC1 stimulation sustains recycling and lysosomal degradation of TCRζ, impacting the sensitivity of such T cells to antigen stimulation. Compensatory upregulation of the Fcε receptor type Iγ chain (FcεRIγ) and recruitment of the tyrosine-protein kinase Syk then mediates enhanced calcium fluxing, prolonging T cells activation and amplifying clonal expansion [58] (Figure 2). mTORC1 hyperactivation has also been implicated in IL-4 and IL-17 production, negatively impacting the population of regulatory T cells [59]. The concept that mTORC1-imposed oxidative stress is a key pathogenic pathway in SLE has been translated into clinical medicine (Figure 2). Treatment with *N*-acetylcysteine, a precursor of glutathione, has been shown to successfully block mTORC signaling and improve disease outcomes in both mouse models and SLE patients [60,61]. Along the same line, treatment with rapamycin, an inhibitor of the mTOR pathway, has also been reported to have beneficial effects in the management of SLE patients and alleviated disease progression [62].

In contrast to the disease-promoting role of mitochondria in driving T cell hyperreactivity, recent data have linked SLE susceptibility to mitochondrial failure. Patients with SLE usually present with decreased levels of complement and individuals born with deficiency of the complement component 1q (C1q) are at high risk for SLE [63]. In a paradigm-shifting study, Botto and colleagues have mechanistically connected C1q deficiency and abnormal adaptive immunity in SLE. Specifically, they found that C1q, but not C3, is capable to restrain the response to self-antigens by modulating the mitochondrial metabolism in CD8^+^ T cells [64]. It has been known for two decades that C1q, when bound to its cognate cell surface receptor p32, translocates into the mitochondrial matrix, where it stimulates mitochondrial function [65]. While T effector cells rely mostly on energy production through glycolysis, T memory cells require mitochondrial energy supply to support their biosynthetic and bioenergetic needs. In the Botto et al study, lack of C1q disfavored the survival of long-lived CD8 memory T cells and instead promoted the differentiation of short-lived, pro-inflammatory CD8 T cells [66]. Consequently, C1q-deficient mice were prone to produce autoantibodies and develop renal inflammation. These data directly link C1q deficiency, insufficient mitochondrial stimulation and tissue inflammation. Notably, the effect of C1q appeared to be relevant to regulate CD8, but not CD4 function, a deviation from the traditional paradigm that assumes that the major drivers of autoantibodies are CD4 helper T cells.

## LIPID METABOLISM ABNORMALITY IN RA AND SLE T CELLS

T cells are distinguished from other immune cell types by their enormous capacity to proliferate. The building of biomass is only possible through the well-tuned and dynamic utilization of energy sources and the proper assignment of energy carriers to either ATP generation or the production of biosynthetic precursors. Producing the membranes for intracellular organelles and the plasma membrane needed for daughter cells imposes high requirement for lipids. In the current paradigm, extracellular lipid supply is not considered a key element to drive uncommitted T cells towards lineage specification for Th1, Tfh or Th17 effector cells. However, intracellular lipid metabolism appears critical for pro-inflammatory T cell function, given the core role lipids play in the membrane systems. Support for the concept that fatty acids increase the propensity towards inflammatory activity comes from studies in obesity. Here, the metabolic stress induced by obesity has been reported to shift T cell differentiation towards the pro-inflammatory effector phenotype [67].

**Figure 3.**
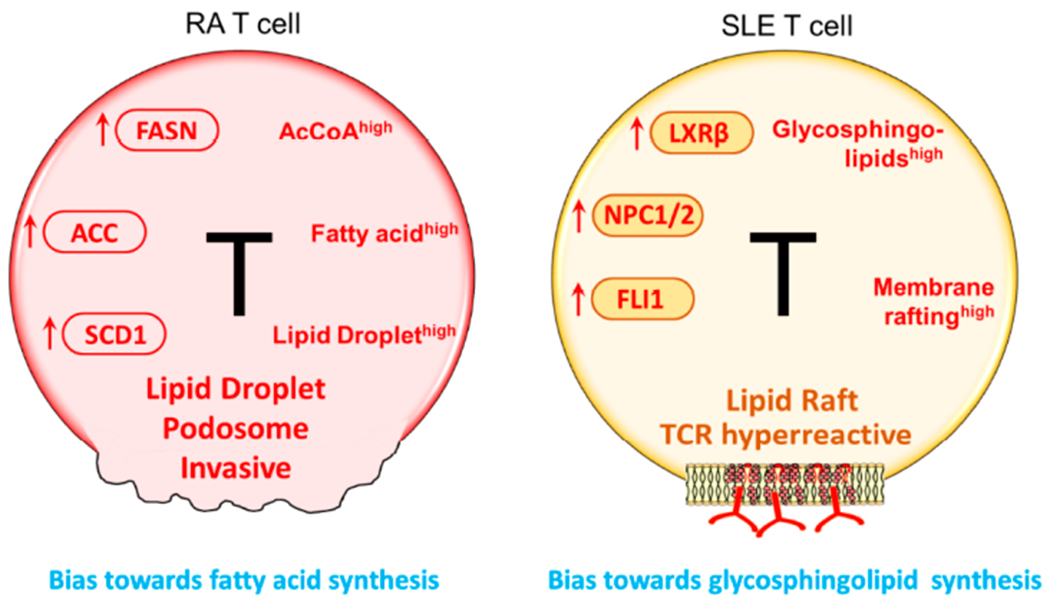
Lipid metabolism in RA and SLE T cells. Despite their ATP^low^ status, RA T cells activate the lipogenesis machinery. Enzymes supporting de novo fatty acid synthesis, including FASN, ACC, and SCD1 are all upregulated. Abundancy in intracellular AcCoA and NADPH facilitate the shift towards lipid generation. As a result, lipid droplets accumulate in the cytoplasm where they serve as a reservoir of biosynthetic activity. Equipped with surplus lipids, RA T cells tend to form membrane extensions; podosome-like structure that enable T cells to invade into matrix and promote tissue inflammation. In SLE T cells, glycosphingolipids (GSLs) synthesis is enhanced. Oversupply in GSLs changes the plasma membrane composition and structure, promoting lipid rafts formation. One outcome of membrane rafting is the enhancement of TCR signaling, enabled by easing the recruitment of lipid raft-associated signaling mediators, such as the proteins of the TCR complex and downstream signaling molecules. FASN, fatty acid synthase; ACC, acetyl-CoA carboxylase; SCD1, stearoyl-CoA desaturase; TCR, T cell receptor.

Compared to healthy T cells, RA T cells upregulate a broad, yet selective, lipogenic gene program focused on fatty acid (FA) synthesis. Acetyl-CoA carboxylase 1 (ACACA), fatty acid synthase (FASN), stearoyl-CoA desaturase (SCD), and the fatty acid-coenzyme A ligase family are critical enzymes in de novo fatty acid synthesis and elongation and all of them are upregulated in RA T cells as they differentiate into effector cells [68] (Figure 3). Fatty acid synthesis is further facilitated by the availability of NADPH produced in the PPP and high levels of intracellular acetyl-CoA [69]. Although mitochondrial fatty acid oxidation has not been investigated in detail, given the low mitochondrial activity and low ATP output [39], it is reasonable to predict insufficient fatty acid utilization in RA T cells. Declining lipolysis would obviously further increase fatty acid accumulation. In line with these data, both ex vivo-stimulated T cells and synovial T cells from RA patients deposit excess fatty acids as cytoplasmic lipid droplets (Figure 3). This metabolic deviation is closely linked to an upregulated locomotion program of RA T cells [68]. The movement of T cells migrating through extracellular space is distinct from that of myeloid cells. T cells utilize amoeba-like locomotion, constantly changing their shape as they edge along collagen fibrils. T cells do not degrade extracellular matrix and thus must squeeze through preexisting matrix gaps [70]. Thus, T cell motility depends on cellular polarization, in which the cytoskeleton is reoriented and dynamic cell protrusions are formed. Compared with healthy T cells, RA T cells are more efficient in entering the collagen matrix and are faster in penetrating into deeper layers. RA-specific changes in T cell locomotion have been successfully captured in transwell chambers [68]. Inhibition of lipogenesis has been reported to correct the hypermigratory properties of RA T cells and effectively suppresses synovitis in a human synovium-chimeric mouse model. It is not surprising that the T cell motility machinery is integrated with abnormal lipid metabolism, as the turnover of membrane structure in migrating T cells largely relies on lipid availability. Accordingly, in T cells with lipid droplet storage, plasma membranes built invasive membrane ruffles. Molecular studies have revealed that formation of membrane protrusions occurs through the co-localization of the cytoskeletal marker F-actin and the membrane marker cortactin [71,72], reminiscent of podosomes utilized by tumor cells to spread and metastasize [73] (Figure 3).

In this context, it is remarkable that rerouting of the glycolytic machinery will eventually alter lipogenesis. This is exemplified in RA T cells, which lack PFKFB3 and have a low yield of pyruvate, yet upregulate lipogenic enzymes [68]. Mechanistically, enhanced fatty acid synthesis was linked to induction of a gene module encompassing a set of motility-associated genes, including TKS5, a scaffolding protein localized at the plasma membrane and important for the formation of invasive membrane ruffles [74]. Upregulation of TKS5 transcription in RA T cells is a prerequisite for tissue invasiveness [68]. Thus, lipid metabolism is firmly integrated into disease-relevant effector functions.

Lupus T cells are more sensitive to T cell antigen receptor (TCR) stimulation, with a decreased threshold of activation required to induce intracytoplasmic calcium (Ca^2+^) fluxing [75]. Glycosphingolipids (GSLs) and cholesterol partner to form lipid rafts in plasma membranes and are important in regulating TCR signaling, as such specialized membrane structures support the formation of the T cell receptor signalosome [76,77]. Compared with healthy controls, CD4 T cells from SLE patients support increased synthesis of GSLs [78] (Figure 3). One possible mechanism is the elevation of the lipid-responsive nuclear receptor LXRβ, together with its target genes NPC1 and NPC2 (Figure 3). Increased GSLs internalization and recycling has been described for SLE CD4 T cells, supporting the building of plasma membranes with distinct lipid composition [78]. Inhibition of GSLs synthesis beneficially affected signaling defects and the hyperproliferative phenotype in T cells from SLE patients. Friend leukemia virus integration 1 (LLI1), an ETS family transcription factor, is upregulated in SLE T cells [79,80], and has also been reported to interfere with GSLs metabolism and TCR signaling (Figure 3). Cholesterol, on the other hand, has not been linked to autoimmune T cell functions. However, statins have been shown to regulate the Th17/Treg balance and could have beneficial effects in several autoimmune diseases including multiple sclerosis, RA and lupus [81].

## ENERGY SENSING IN RA AND SLE T CELLS

During their life cycle T cells need to adapt to changing tissue environments, periods of high proliferative stress, clonal expansion and clonal retraction, episodes of quiescence and differentiation into fast and highly functional effector cells. A key element in the adaptability of T cells is their highly effective sensing of intracellular energy resources and metabolic activities. The AMP-activated protein kinase (AMPK) serves as the main energy sensor in almost all eukaryotic cells [82]. Canonically, AMPK monitors AMP/ATP and ADP/ATP ratios and restores energy homeostasis by enhancing ATP-producing catabolic processes and inhibiting energy-utilizing anabolic activities. Besides directly measuring the availability of ATP, AMPK is capable of assessing the metabolic status through several nucleotide-independent pathways [82]. As a trimeric complex, AMPK consists of a catalytic subunit (α subunit) and two regulatory subunits (β and γ subunits). Whatever upstream signal or regulation is employed, AMPK activation is initiated by the phosphorylation of the a subunit, and AMPK activation has been tightly linked to the pool of membrane-associated AMPK, rather than unbound cytosolic AMPK [83–85].

**Figure 4.**
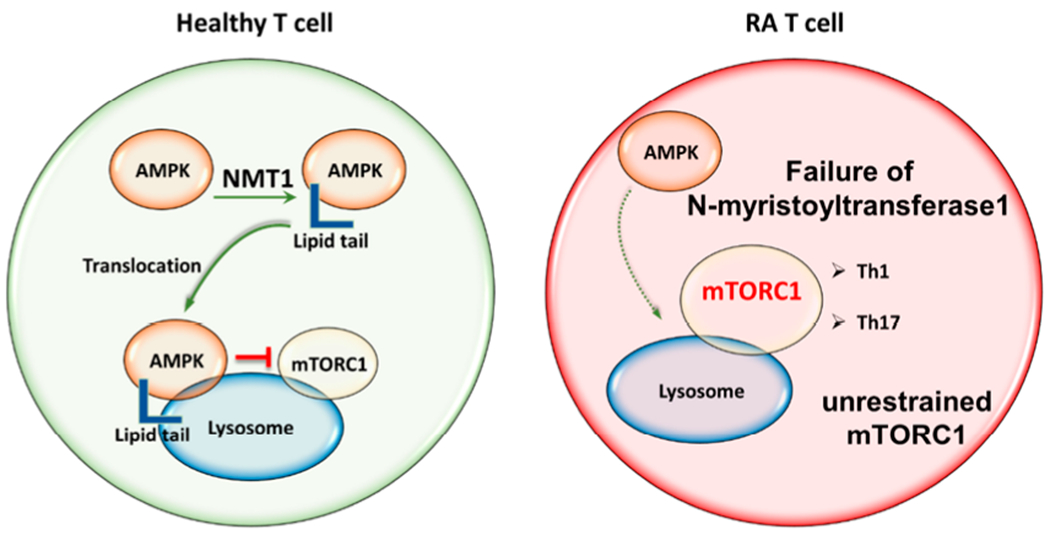
Misrouting of AMPK in RA T cells. AMPK is the major sensor of the cell’s energy status and co-ordinates energy reserves with biosynthetic activity by regulating mTORC1. AMPK activation occurs on the surface of the lysosome, where it co-localizes with mTORC1. Activated AMPK signals a need for catabolic activity and suppresses mTORC1. The recruitment of AMPK to the lysosomal surface requires the addition of a myristic acid lipid tail, that enables anchoring in the lysosomal membrane. Protein myristoylation is mediated by *N*-myristoyltransferase-1 (NMT1). Failure of NMT1 in RA T cells alters the intracellular trafficking of AMPK and prevents translocation to the cytoplasmic surface of the lysosome. One of the outcomes is unrestrained mTORC1 activation and a commitment of RA T cells to cellular proliferation and anabolic metabolism. AMPK, AMP-activated protein kinase; mTORC1, mechanistic target of rapamycin complex 1; NMT1, *N*-Myristoyltransferase-1.

An open question has been how T cells from RA patients, given their diminished glycolysis and low ATP production [86], monitor and adjust their proliferative and biosynthetic activities as well as their commitment to the production of effector molecules. Initial studies revealed that AMPK activity was significantly inhibited in RA T cells [87], despite the low ATP conditions and high cellular turnover. Essentially, the cellular rheostat connecting energy supply and utilization is no longer intact in these T cells. Inappropriately low mobilization of AMPK signaling did however provide an explanation for several features of RA T cells: reduced mitochondrial activity and reduced ATP synthesis; low intracellular ROS concentrations; shunting of glucose into the PPP instead of into pyruvate generation; inability to metabolize surplus lipids, and persistently high activation of mTORC1. In a series of molecular and cell fractionation experiments, the mechanism underlying defective AMPK activation has been solved [87]. Essentially, RA T cells maintain AMPK in the unbound, cytosolic pool and fail to translocate the protein to the lysosomal surface (Figure 4). The misplacement of AMPK is a consequence of insufficient *N*-myristoylation of the β-subunit of AMPK (AMPKβ). Lacking a myristate tail, AMPK cannot be recruited to the lysosomal membrane and RA T cells fail to form the v-ATPase-Ragulator-AXIN/LKB1-AMPK super-complex, where AMPKα-Thr172 is phosphorylated by LKB1 [88]. An important consequence of AMPK misplacement is the disruption of AMPK-mTORC1 cross-regulation. Lysosomal AMPK suppresses activation of co-localized mTORC1, shifting the cells towards anabolic and away from catabolic activity [88] (Figure 4). Thus, RA T cells fail to prevent mTORC1 activation and several functional activities have been assigned to persistence of mTORC1 activity [87] (Figure 4). In human synovium-mouse chimeras, blocking mTORC1 activity via rapamycin therapy was highly effective to suppress synovitis [87]. The molecular defect causing AMPK mis-trafficking towards the unbound cytosolic pool has been identified. Addition of the myristic acid lipid tail to AMPK requires the enzyme ΑΓ-myristoyltransferase-1 (NMT1) [87]. In RA T cells, this transferase is suppressed through a post-transcriptional mechanism (Figure 4). Replenishing NMT1 in RA T cells rescued AMPK activation and corrected the pro-inflammatory properties of these T cells. Interestingly, besides modulating mTORC1 signal, recent studies have shown that activation of AMPK can limit JAK-STAT-dependent signaling pathways, a major driver of RA-dependent events [89].

Inappropriate activation of mTORC1 is a shared feature of RA and SLE T cells, compatible with the notion that distinct metabolic abnormalities can convene to promote disease-relevant immunity. In T cells from SLE patients, mTORC1 hyper-activation has been identified as the main driver of T cell dysfunction. In clinical studies, the mTOR inhibitor rapamycin has shown promise in ameliorating SLE disease activity; presumably, by correcting pro-inflammatory T-cell lineage specification [62,90]. While inappropriate mTORC1 activity in SLE T cells has been attributed to mitochondrial stress and excess production of mitochondrial ROS, the role of AMPK in controlling mTOR signaling has been insufficiently examined. The AMPK activation status has not been systemically studied in SLE T cells, but indirect evidence, such as the high level of catalytic metabolism, the enhanced mitochondrial biogenesis and the intensified mitochondrial respiration, would all suggest that AMPK is in hyperdrive. Metformin, a known activator of AMPK and a first-line pharmacological therapy for type II diabetes mellitus, has been tested as an immunomodulator in SLE [25,91], but more definitive data are needed to gauge the potential role of targeting AMPK in treating SLE patients.

## CONCLUSIONS

**Figure 5.**
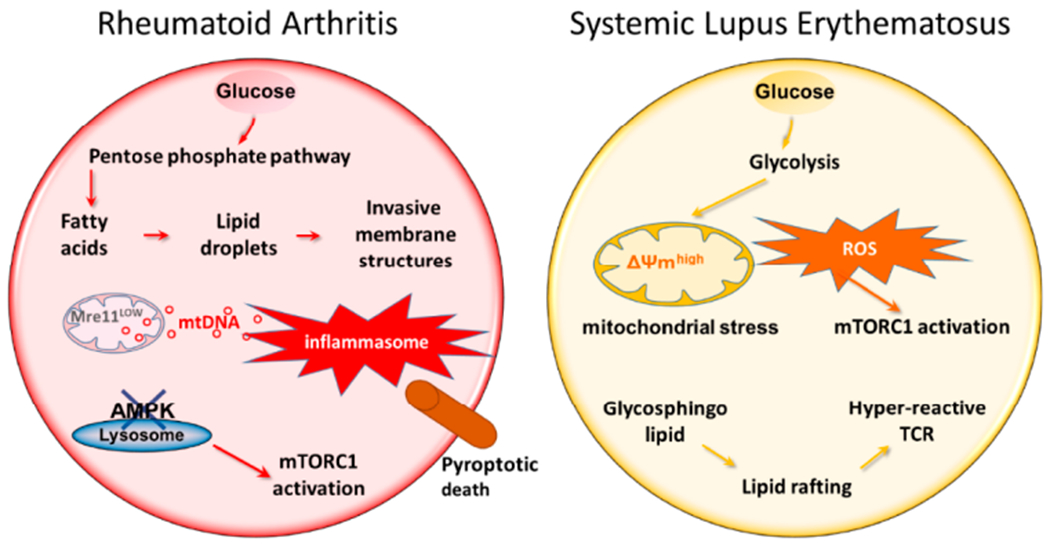
Comparison of the metabolic signatures in T cells from RA and SLE patients.

The recognition that the utilization of energy carriers and the biosynthetic activity for biomass generation are critical elements in determining cellular fate decisions and cellular differentiation has opened the emerging field of immunometabolism. Metabolic control of cellular behavior may be particularly important for inflammatory cells, such as autoreactive T cells that drive chronic tissue inflammation in autoimmune disease. T cells from patients with the autoimmune diseases RA and SLE display distinct metabolic signatures, and in both cases, metabolic deviations have been linked to abnormal cellular behavior (Figure 5). Accumulated data allow one to draw a metabolic landscape, in which metabolic interference may become a novel immunomodulatory therapy.

1. In RA patients, CD4 T cells shift glucose towards the pentose phosphate pathway (PPP), favor anabolic metabolism and support a cell building program. This abnormality affects the naive CD4 T cell population and may be instrumental in the initial events leading to the loss of self-tolerance in RA patients.
2. In SLE patients, CD4 T cells prefer glycolytic breakdown of glucose to fuel T cell effector activity, especially the induction and maintenance of Th17 cells.
3. T cells from RA patients have a defect in mitochondrial function. Due to insufficient repair of mitochondrial DNA, mitochondrial oxygen consumption is low and ATP generation is impaired. Damaged mitochondrial DNA escapes into the cytoplasm and is recognized as a DAMP. Inflammasome assembly and caspase-1 activation result in pyroptotic T cell death and trigger robust tissue inflammation.
4. T cells from SLE patients are rich in mitochondria and rich in mitochondrial ROS. In these oxidatively stressed T cells, mTORC1 is activated and T cell receptor signaling is enhanced, resulting in T cell hyperactivity and tissue inflammation.
5. In T cells of RA patients, sensing of intracellular ATP levels is disrupted due to misplacement of the energy sensor AMPK. Under ATP low conditions, AMPK is recruited to the cytoplasmic surface of lysosomes, where the kinase blocks mTORC1 activation and promotes catabolic pathways. Failure of N-myristoyltransferase-1 in RA T cells leaves AMPK without a lipid tail and misroutes the protein to the fraction of cytoplasmic unbound AMPK.
6. A shared metabolic vulnerability in RA and SLE T cells is the persistent activation of mTORC1.

## References

[1] Aviña-Zubieta JA, Choi HK, Sadatsafavi M, Etminan M, Esdaile JM, Lacaille D. Risk of cardiovascular mortality in patients with rheumatoid arthritis: a meta-analysis of observational studies. Arthritis Rheum. 2008;59:1690–17. doi: 10.1002/art.24092 19035419

[2] Maradit-Kremers H, Nicola PJ, Crowson CS, Ballman KV, Gabriel SE. Cardiovascular death in rheumatoid arthritis: a population-based study. Arthritis Rheum. 2005;52:722–32. doi: 10.1002/art.20878 15751097

[3] Esdaile JM, Abrahamowicz M, Grodzicky T, Li Y, Panaritis C, du Berger R, Traditional Framingham risk factors fail to fully account for accelerated atherosclerosis in systemic lupus erythematosus. Arthritis Rheum. 2001;44:2331–7. 11665973 10.1002/1529-0131(200110)44:10<2331::aid-art395>3.0.co;2-i

[4] Deane KD, El-Gabalawy H. Pathogenesis and prevention of rheumatic disease: focus on preclinical RA and SLE. Nat Rev Rheumatol. 2014;10:212–28. doi: 10.1038/nrrheum.2014.6 24514912 PMC4090326

[5] Weyand CM, Goronzy JJ. Immunometabolism in early and late stages of rheumatoid arthritis. Nat Rev Rheumatol. 2017;13:291–301. doi: 10.1038/nrrheum.2017.49 28360422 PMC6820517

[6] Chemin K, Gerstner C, Malmstrom V. Effector Functions of CD4^+^ T Cells at the Site of Local Autoimmune Inflammation-Lessons From Rheumatoid Arthritis. Front Immunol. 2019;10:353. doi: 10.3389/fimmu.2019.00353 30915067 PMC6422991

[7] Howie D, Ten Bokum A, Necula AS, Cobbold SP, Waldmann H. The Role of Lipid Metabolism in T Lymphocyte Differentiation and Survival. Front Immunol 2017;8:1949. doi: 10.3389/fimmu.2017.01949 29375572 PMC5770376

[8] Macintyre AN, Gerriets VA, Nichols AG, Michalek RD, Rudolph MC, Deoliveira D, The glucose transporter Glut1 is selectively essential for CD4 T cell activation and effector function. Cell Metab. 2014;20:61–72. doi: 10.1016/j.cmet.2014.05.004 24930970 PMC4079750

[9] Wang R, Dillon CP, Shi LZ, Milasta S, Carter R, Finkelstein D, The transcription factor Myc controls metabolic reprogramming upon T lymphocyte activation. Immunity. 2011;35:871–82. doi: 10.1016/j.immuni.2011.09.021 22195744 PMC3248798

[10] Michalek RD, Gerriets VA, Jacobs SR, Macintyre AN, Maclver NJ, Mason EF, Cutting edge: distinct glycolytic and lipid oxidative metabolic programs are essential for effector and regulatory CD4^+^ T cell subsets. J Immunol. 2011;186:3299–303. doi: 10.4049/jimmunol.l003613 21317389 PMC3198034

[11] Desdin-Mico G, Soto-Heredero G, Mittelbrunn M. Mitochondrial activity in T cells. Mitochondrion. 2018;41:51–7. doi: 10.1016/j.mito.2017.10.006 29032101

[12] Yang Z, Fujii H, Mohan SV, Goronzy JJ, Weyand CM. Phosphofructokinase deficiency impairs ATP generation, autophagy, and redox balance in rheumatoid arthritis T ceils. J Exp Med. 2013;210:2119–34. doi: 10.1084/jem.20130252 24043759 PMC3782046

[13] Weyand CM, Goronzy JJ. Immunometabolism in the development of rheumatoid arthritis. Immunol Rev. 2020 3;294(l):177–87. doi: 10.1111/imr.l2838 31984519 PMC7047523

[14] Yang Z, Matteson EF, Goronzy JJ, Weyand CM. T-cell metabolism in autoimmune disease. Arthritis Res Ther. 2015;17:29. doi: 10.1186/sl3075-015-0542-4 25890351 PMC4324046

[15] Weyand CM, Zeisbrich M, Goronzy JJ. Metabolic signatures of T-cells and macrophages in rheumatoid arthritis. Curr Opin Immunol. 2017;46:112–20. doi: 10.1016/j.coi.2017.04.010 28538163 PMC5554742

[16] Yang Z, Shen Y, Oishi H, Matteson EL, Tian L, Goronzy JJ, Restoring oxidant signaling suppresses proarthritogenic T cell effector functions in rheumatoid arthritis. Sci Transl Med. 2016;8(331):331ra38. doi: 10.1126/scitranslmed.aad7151 PMC5074090 27009267

[17] Zeisbrich M, Yanes RE, Zhang H, Watanabe R, Li Y, Brosig L, Hypermetabolic macrophages in rheumatoid arthritis and coronary artery disease due to glycogen synthase kinase 3b inactivation. Ann Rheum Dis. 2018;77:1053–62. doi: 10.1136/annrheumdis-2017-212647 29431119 PMC6589337

[18] Petrasca A Phelan JJ, Ansboro S, Veale DJ Fearon U, Fletcher JM. Targeting bioenergetics prevents CD4 T cell-mediated activation of synovial fibroblasts in rheumatoid arthritis. Rheumatology. 2020 2 11;kez682. doi: 10.1093/rheumatology/kez682 32047926

[19] Biniecka M, Canavan M, McGarry T, Gao W, McCormick J, Cregan S, Dysregulated bioenergetics: a key regulator of joint inflammation. Ann Rheum Dis. 2016;75:2192–200. doi: 10.1136/annrheumdis-2015-208476 27013493 PMC5136702

[20] Treuhaft PS, MCCarty DJ. Synovial fluid pH, lactate, oxygen and carbon dioxide partial pressure in various joint diseases. Arthritis Rheum. 1971;14:475–84. doi: 10.1002/art.l780140407 5564921

[21] Pucino V, Certo M, Bulusu V, Cucchi D, Goldmann K, Pontarini E, Lactate Buildup at the Site of Chronic Inflammation Promotes Disease by Inducing CD4(+) T Cell Metabolic Rewiring. Cell Metab. 2019;30:1055–74.el058. doi: 10.1016/j.cmet.2019.10.004 31708446 PMC6899510

[22] Haas R, Smith J, Rocher-Ros V, Nadkarni S, Montero-Melendez T, D’Acquisto F, Lactate Regulates Metabolic and Pro-inflammatory Circuits in Control of T Ceil Migration and Effector Functions. PLoS Biol. 2015;13:el002202 10.1371/journal.pbio.l002202 PMC4504715 26181372

[23] Panneton V, Bagherzadeh Yazdchi S, Witalis M, Chang J, Suh WK. ICOS Signaling Controls Induction and Maintenance of Collagen-Induced Arthritis. J Immunol. 2018;200:3067–76. doi: 10.4049/jimmunol.1701305 29581356

[24] Yin Y, Choi SC, Xu Z, Perry DJ, Seay H, Croker BP, Normalization of CD4^+^ T cell metabolism reverses lupus. Sci Transl Med. 2015;7:274ra218. doi: 10.1126/scitranslmed.aaa0835 PMC5292723 25673763

[25] Yin Y, Choi S-C, Xu Z, Zeumer L, Kanda N, Croker BP, Glucose Oxidation Is Critical for CD4^+^ T Cell Activation in a Mouse Model of Systemic Lupus Erythematosus. J Immunol. 2016;196:80–90. doi: 10.4049/jimmunol.1501537 26608911 PMC4684991

[26] Wahl DR, Petersen B, Warner R, Richardson BC, Glick GD, Opipari AW. Characterization of the metabolic phenotype of chronically activated lymphocytes. Lupus. 2010;19:1492–501. doi: 10.1177/0961203310373109 20647250

[27] Perl A, Hanczko R, Lai Z-W, Oaks Z, Kelly R, Borsuk R, Comprehensive metabolome analyses reveal A-acetylcysteine-responsive accumulation of kynurenine in systemic lupus erythematosus: implications for activation of the mechanistic target of rapamycin. Metabolomics. 2015;11:1157–74. doi: 10.1007/s11306-015-0772-0 26366134 PMC4559110

[28] Kim SJ, Lee K, Diamond B. Follicular Helper T Cells in Systemic Lupus Erythematosus. Front Immunol. 2018;9:1793. doi: 10.3389/fimmu.2018.01793 30123218 PMC6085416

[29] Choi SC, Titov AA, Abboud G, Seay HR, Brusko TM, Roopenian DC, Inhibition of glucose metabolism selectively targets autoreactive follicular helper T cells. Nat Commun. 2018;9:4369. doi: 10.1038/s41467-018-06686-0 30348969 PMC6197193

[30] Crispín JC, Oukka M, Bayliss G, Cohen RA, Van Beek CA, Stillman IE, Expanded double negative T cells in patients with systemic lupus erythematosus produce IL-17 and infiltrate the kidneys. J Immunol. 2008;181:8761–6. doi: 10.4049/jimmunol.181.12.8761 19050297 PMC2596652

[31] Pisitkun P, Ha H-L, Wang H, Claudio E, Tivy CC, Zhou H, Interleukin-17 cytokines are critical in development of fatal lupus glomerulonephritis. Immunity. 2012;37:1104–15. doi: 10.1016/j.immuni.2012.08.014 23123062 PMC3594848

[32] Yoshida N, Comte D, Mizui M, Otomo K, Rosetti F, Mayadas TN, ICER is requisite for Th17 differentiation. Nat Commun. 2016;7:12993. doi: 10.1038/ncomms12993 27680869 PMC5056420

[33] Kono M, Yoshida N, Maeda KM, Skinner NE, Pan W, Kyttaris VC, Pyruvate dehydrogenase phosphatase catalytic subunit 2 limits Th17 differentiation. Proc Natl Acad Sci U S A. 2018;115:9288–93. doi: 10.1073/pnas.1805717115 30150402 PMC6140513

[34] Koetz K, Bryl E, Spickschen K, O’Fallon WM, Goronzy JJ, Weyand CM. T cell homeostasis in patients with rheumatoid arthritis. Proc Natl Acad Sci U S A. 2000;97:9203–8. doi: 10.1073/pnas.97.16.9203 10922071 PMC16846

[35] Schonland SO, Lopez C, Widmann T, Zimmer J, Bryl E, Goronzy JJ, Premature telomeric loss in rheumatoid arthritis is genetically determined and involves both myeloid and lymphoid cell lineages. Proc Natl Acad Sci U S A. 2003;100:13471–6. doi: 10.1073/pnas.2233561100 14578453 PMC263838

[36] Goronzy JJ, Weyand CM. Mechanisms underlying T cell ageing. Nat Rev Immunol. 2019;19:573–83. doi: 10.1038/s41577-019-0180-1 31186548 PMC7584388

[37] Li Y, Goronzy JJ, Weyand CM. DNA damage, metabolism and aging in pro-inflammatory T cells: Rheumatoid arthritis as a model system. Exp Gerontol. 2018;105:118–27. doi: 10.1016/j.exger.2017.10.027 29101015 PMC5871568

[38] Weyand CM, Yang Z, Goronzy JJ. T-cell aging in rheumatoid arthritis. Curr Opin Rheumatol. 2014;26:93–100. doi: 10.1097/BC>R.0000000000000011 24296720 PMC3984035

[39] Li Y, Shen Y, Jin K, Wen Z, Cao W, Wu B, The DNA Repair Nuclease MRE11A Functions as a Mitochondrial Protector and Prevents T Cell Pyroptosis and Tissue Inflammation. Cell Metab. 2019;30:477–92.e476. doi: 10.1016/j.cmet.2019.06.016. 31327667 PMC7093039

[40] Li Y, Shen Y, Hohensinner P, Ju J, Wen Z, Goodman SB, Deficient Activity of the Nuclease MRE11A Induces T Cell Aging and Promotes Arthritogenic Effector Functions in Patients with Rheumatoid Arthritis. Immunity. 2016;45:903–16. doi: 10.1016/j.immuni.2016.09.013 27742546 PMC5123765

[41] Doitsh G, Galloway NL, Geng X, Yang Z, Monroe KM, Zepeda O, Cell death by pyroptosis drives CD4 T-cell depletion in HIV-1 infection. Nature. 2014;505:509–14. doi: 10.1038/naturel2940 24356306 PMC4047036

[42] Kahlenberg JM, Kaplan MJ. The inflammasome and lupus: another innate immune mechanism contributing to disease pathogenesis? Curr Opin Rheumatol. 2014;26:475–81. doi: 10.1097/BOR.0000000000000088 24992143 PMC4153426

[43] Shen HH, Yang YX, Meng X, Luo XY, Li XM, Shuai ZW, NLRP3: A promising therapeutic target for autoimmune diseases. Autoimmun Rev. 2018;17:694–702. doi: 10.1016/j.autrev.2018.01.020 29729449

[44] Ikeda S, Saijo S, Murayama MA, Shimizu K, Akitsu A, Iwakura Y. Excess IL-1 signaling enhances the development of Th17 cells by downregulating TGF-beta-induced Foxp3 expression. J Immunol. 2014;192:1449–58. doi: 10.4049/jimmunol.l300387 24431229

[45] Mailer RK, Joly A-L, Liu S, Elias S, Tegner J, Andersson J, IL-lbeta promotes Th17 differentiation by inducing alternative splicing of FOXP3. Sci Rep. 2015;5:14674. doi: 10.1038/srepl4674 26441347 PMC4593960

[46] Yang CA, Huang ST, Chiang BL. Sex-dependent differential activation of NLRP3 and AIM2 inflammasomes in SLE macrophages. Rheumatology (Oxford). 2015;54:324–31. doi: 10.1093/rheumatology/keu318 25161312

[47] Westerterp M, Gautier EL, Ganda A, Molusky MM, Wang W, Fotakis P, Cholesterol Accumulation in Dendritic Cells Links the Inflammasome to Acquired Immunity. Cell Metab. 2017;25:1294–304.el296. doi: 10.1016/j.cmet.2017.04.005 28479366 PMC5514787

[48] Maczynska I, Millo B, Ratajczak-Stefańska V, Maleszka R, Szych Z, Kurpisz M, Proinflammatory cytokine (IL-lbeta, IL-6, IL-12, IL-18 and TNF-alpha) levels in sera of patients with subacute cutaneous lupus erythematosus (SCLE). Immunol Lett. 2006;102:79–82. doi: 10.1016/j.imlet.2005.08.001 16154204

[49] Dinarello CA, Novick D, Kim S, Kaplanski G. Interleukin-18 and IL-18 binding protein. Front Immunol. 2013;4:289. doi: 10.3389/fimmu.2013.00289 24115947 PMC3792554

[50] Lech M, Lorenz G, Kulkarni OP, Grosser MOO, Stigrot N, Darisipudi MN, NLRP3 and ASC suppress lupus-like autoimmunity by driving the immunosuppressive effects of TGF-beta receptor signalling. Ann Rheum Dis. 2015;74:2224–35. doi: 10.1136/annrheumdis-2014-205496 25135254

[51] Gergely P Jr, Grossman C, Niland B, Puskas F, Neupane H, AHam F, Mitochondrial hyperpolarization and ATP depletion in patients with systemic lupus erythematosus. Arthritis Rheum. 2002;46:175–90. 11817589 10.1002/1529-0131(200201)46:1<175::AID-ART10015>3.0.CO;2-H PMC4020417

[52] Perl A Oxidative stress in the pathology and treatment of systemic lupus erythematosus. Nat Rev Rheumatol. 2013;9:674–86. doi: 10.1038/nrrheum.2013.147 24100461 PMC4046645

[53] Nagy G, Barcza M, Gonchoroff N, Phillips PE, Perl A. Nitric oxide-dependent mitochondrial biogenesis generates Ca^2+^ signaling profile of lupus T cells. J Immunol. 2004;173:3676–83. doi: 10.4049/jimmunol.l73.6.3676 15356113 PMC4034140

[54] Doherty E, Oaks Z, Perl A. Increased mitochondrial electron transport chain activity at complex I is regulated by ΑΓ-acetylcysteine in lymphocytes of patients with systemic lupus erythematosus. Antioxid Redox Signal. 2014;21:56–65. doi: 10.1089/ars.2013.5702 24673154 PMC4048573

[55] Desai BN, Myers BR, Schreiber SL. FKBP12-rapamycin-associated protein associates with mitochondria and senses osmotic stress via mitochondrial dysfunction. Proc Natl Acad Sci U S A. 2002;99:4319–24. doi: 10.1073/pnas.261702698 11930000 PMC123646

[56] Fernandez DR, Telarico T, Bonilla E, Li Q, Banerjee S, Middleton FA, Activation of mammalian target of rapamycin controls the loss of TCRzeta in lupus T cells through HRES-l/Rab4-regulated lysosomal degradation. J Immunol. 2009;182:2063–73. doi: 10.4049/jimmunol.0803600 19201859 PMC2676112

[57] Sabatini DM. Twenty-five years of mTOR: Uncovering the link from nutrients to growth. Proc Natl Acad Sci U S A. 2017;114:11818–25. doi: 10.1073/pnas.1716173114 29078414 PMC5692607

[58] Tsokos GC. Systemic lupus erythematosus. N Engl J Med. 2011;365:2110–21. doi: 10.1056/NEJMrall00359 22129255

[59] Kato H, Perl A. Mechanistic target of rapamycin complex 1 expands Th17 and IL-4^+^ CD4^−^CD8^−^ double-negative T cells and contracts regulatory T cells in systemic lupus erythematosus. J Immunol. 2014;192:4134–44. doi: 10.4049/jimmunol.l301859 24683191 PMC3995867

[60] Lai ZW, Hanczko R, Bonilla E, Caza TN, Clair B, Bartos A, *N*-acetylcysteine reduces disease activity by blocking mammalian target of rapamycin in T cells from systemic lupus erythematosus patients: a randomized, double-blind, placebo-controlled trial. Arthritis Rheum. 2012;64:2937–46. doi: 10.1002/art.34502 22549432 PMC3411859

[61] Suwannaroj S, Lagoo A, Keisler D, McMurray RW. Antioxidants suppress mortality in the female NZB × NZW F1 mouse model of systemic lupus erythematosus (SLE). Lupus. 2001;10:258–65. doi: 10.1191/096120301680416940 11341102

[62] Fernandez D, Bonilla E, Mirza N, Niland B, Perl A. Rapamycin reduces disease activity and normalizes T cell activation-induced calcium fluxing in patients with systemic lupus erythematosus. Arthritis Rheum. 2006;54:2983–8. doi: 10.1002/art.22085 16947529 PMC4034146

[63] Manderson AP, Botto M, Walport MJ. The role of complement in the development of systemic lupus erythematosus. Annu Rev Immunol. 2004;22:431–56. doi: 10.1146/annurev.immunol.22.012703.104549 15032584

[64] Ling GS, Crawford G, Buang N, Bartok I, Tian K, Thielens NM, C1q restrains autoimmunity and viral infection by regulating CD8(+) T cell metabolism. Science. 2018;360:558–63. doi: 10.1126/science.aao4555 29724957 PMC6545171

[65] Yagi M, Uchiumi T, Takazaki S, Okuno B, Nomura M, Yoshida S-i, p32/gC1qR is indispensable for fetal development and mitochondrial translation: importance of its RNA-binding ability. Nucleic Acids Res. 2012;40:9717–37. doi: 10.1093/nar/gks774 22904065 PMC3479211

[66] Weyand CM, Goronzy JJ. A Mitochondrial Checkpoint in Autoimmune Disease. Cell Metab. 2018;28:185–6. doi: 10.1016/j.cmet.2018.07.014 30089238 PMC6487495

[67] Mauro C, Smith J, Cucchi D, Coe D, Hongmei Fu H, Bonacina F, Obesity-Induced Metabolic Stress Leads to Biased Effector Memory CD4(+) T Cell Differentiation via PI3K p110delta-Akt-Mediated Signals. Cell Metab. 2017;25:593–609. doi: 10.1016/j.cmet.2017.01.008 28190771 PMC5355363

[68] Shen Y, Wen Z, Li Y, Matteson EL, Hong J, Goronzy JJ, Metabolic control of the scaffold protein TKS5 in tissue-invasive, proinflammatory T cells. Nat Immunol. 2017;18:1025–34. doi: 10.1038/ni.3808 28737753 PMC5568495

[69] Weyand CM, Shen Y, Goronzy JJ. Redox-sensitive signaling in inflammatory T cells and in autoimmune disease. Free Radic Biol Med. 2018;125:36–43. doi: 10.1016/j.freeradbiomed.2018.03.004 29524605 PMC6128787

[70] Wolf K, Muller R, Borgmann S, Brocker EB, Friedl P. Amoeboid shape change and contact guidance: T-lymphocyte crawling through fibrillar collagen is independent of matrix remodeling by MMPs and other proteases. Blood. 2003;102:3262–9. doi: 10.1182/blood-2002-12-3791 12855577

[71] Schnoor M, Stradal TE, Rottner K. Cortactin: Cell Functions of A Multifaceted Actin-Binding Protein. Trends Cell Biol. 2018;28:79–98. doi: 10.1016/j.tcb.2017.10.009 29162307

[72] Beaty BT, Condeelis J. Digging a little deeper: the stages of invadopodium formation and maturation. Eur J Cell Biol. 2014;93:438–44. doi: 10.1016/j.ejcb.2014.07.003 25113547 PMC4262566

[73] Eddy RJ, Weidmann MD, Sharma VP, Condeelis JS. Tumor Cell Invadopodia: Invasive Protrusions that Orchestrate Metastasis. Trends Cell Biol. 2017;27:595–607. doi: 10.1016/j.tcb.2017.03.003 28412099 PMC5524604

[74] Courtneidge SA. Cell migration and invasion in human disease: the Tks adaptor proteins. Biochem Soc Trans. 2012;40:129–32. doi: 10.1042/BST20110685 22260678 PMC3425387

[75] Krishnan S, Nambiar MP, Warke VG, Fisher CU, Mitchell J, Delaney N, Alterations in lipid raft composition and dynamics contribute to abnormal T cell responses in systemic lupus erythematosus. J Immunol. 2004;172:7821–31. doi: 10.4049/jimmunol.172.12.7821 15187166

[76] Werlen G, Palmer E. The T-cell receptor signalosome: a dynamic structure with expanding complexity. Curr Opin Immunol. 2002;14:299–305. doi: 10.1016/s0952-7915(02)00339-4 11973126

[77] He HT, Lellouch A, Marguet D. Lipid rafts and the initiation of T cell receptor signaling. Semin Immunol. 2005;17:23–33. doi: 10.1016/j.smim.2004.09.001 15582486

[78] McDonald G, Deepak S, Miguel L, Hall CJ, Isenberg DA, Magee AI, Normalizing glycosphingolipids restores function in CD4^+^ T cells from lupus patients. J Clin Invest. 2014;124:712–24. doi: 10.1172/JCI69571 24463447 PMC3904606

[79] Mathenia J, Reyes-Cortes E, Williams S, Molano I, Ruiz P, Watson DK, Impact of Fli-1 transcription factor on autoantibody and lupus nephritis in NZM2410 mice. Clin Exp Immunol. 2010;162:362–71. doi: 10.1111/j.l365-2249.2010.04245.x 20731671 PMC2996603

[80] Georgiou P, Maroulakou I, Green J, Dantis P, Romanospica V, Kottaridis S, Expression of ets family of genes in systemic lupus erythematosus and Sjogren’s syndrome. Int J Oncol. 1996;9:9–18. 21541474

[81] Ulivieri C, Baldari CT. Statins: from cholesterol-lowering drugs to novel immunomodulators for the treatment of Th17-mediated autoimmune diseases. Pharmacol Res. 2014;88:41–52. doi: 10.1016/j.phrs.2014.03.001 24657239

[82] Garcia D, Shaw RJ. AMPK: Mechanisms of Cellular Energy Sensing and Restoration of Metabolic Balance. Mol Cell. 2017;66:789–800. doi: 10.1016/j.molcel.2017.05.032 28622524 PMC5553560

[83] Liang J, Xu Z-X, Ding Z, Lu Y, Yu Q, Werle KD, Myristoylation confers noncanonical AMPK functions in autophagy selectivity and mitochondrial surveillance. Nat Commun. 2015;6:7926. doi: 10.1038/ncomms8926 26272043

[84] Oakhill JS, Chen Z-P, Scott JW, Steel R, Casteili LA, Ling N, beta-Subunit myristoylation is the gatekeeper for initiating metabolic stress sensing by AMP-activated protein kinase (AMPK). Proc Natl Acad Sci U S A. 2010;107:19237–41. doi: 10.1073/pnas.l009705107 20974912 PMC2984171

[85] Miyamoto T, Rho E, Sample V, Akano H, Magari M, Ueno T, Compartmentalized AMPK signaling illuminated by genetically encoded molecular sensors and actuators. Cell Rep. 2015;11:657–70. doi: 10.1016/j.celrep.2015.03.057 25892241 PMC4417068

[86] Weyand CM, Wu B, Goronzy JJ. The metabolic signature of T cells in rheumatoid arthritis. Curr Opin Rheumatol. 2020;32:159–67. doi: 10.1097/BCIR.0000000000000683 PMC9107323 31895885

[87] Wen Z, Jin K, Shen Y, Yang Z, Li Y, Wu B, IV-myristoyltransferase deficiency impairs activation of kinase AMPK and promotes synovial tissue inflammation. Nat Immunol. 2019;20:313–25. doi: 10.1038/s41590-018-0296-7 30718913 PMC6396296

[88] Zhang CS, Jiang B, Li M, Zhu M, Peng Y, Zhang Y-L, The lysosomal v-ATPase-Ragulator complex is a common activator for AMPK and mTORC1, acting as a switch between catabolism and anabolism. Cell Metab. 2014;20:526–40. doi: 10.1016/j.cmet.2014.06.014 25002183

[89] Speirs C, Williams JJL, Riches K, Salt IP, Palmer TM. Linking energy sensing to suppression of JAK-STAT signalling: A potential route for repurposing AMPK activators? Pharmacol Res. 2018;128:88–100. doi: 10.1016/j.phrs.2017.10.001 29037480

[90] Lai ZW, Kelly R, Winans T, Marchena I, Shadakshari A, Yu J, Sirolimus in patients with clinically active systemic lupus erythematosus resistant to, or intolerant of, conventional medications: a single-arm, open-label, phase 1/2 trial. Lancet. 2018;391:1186–96. doi: 10.1016/S0140-6736(18)30485-9 29551338 PMC5891154

[91] Wang H, Li T, Chen S, Gu Y, Ye S. Neutrophil Extracellular Trap Mitochondrial DNA and Its Autoantibody in Systemic Lupus Erythematosus and a Proof-of-Concept Trial of Metformin. Arthritis Rheumatol. 2015;67:3190–200. doi: 10.1002/art.39296 26245802

